# The PI3K/AKT Pathway Is Activated by HGF in NT2D1 Non-Seminoma Cells and Has a Role in the Modulation of Their Malignant Behavior

**DOI:** 10.3390/ijms21228669

**Published:** 2020-11-17

**Authors:** Luisa Gesualdi, Erica Leonetti, Alessandra Cucina, Bianca Maria Scicchitano, Silvia Sorrentino, Maria Grazia Tarsitano, Andrea Isidori, Mariano Bizzarri, Antonio Filippini, Anna Riccioli, Marcella Cammarota, Vincenzo Gigantino, Giulia Ricci, Angela Catizone

**Affiliations:** 1 Section of Histology and Medical Embryology, Department of Anatomy, Histology, Forensic-Medicine and Orthopedics, “Sapienza” University of Rome, 00161 Rome, Italy; luisa.gesualdi@uniroma1.it (L.G.); erica.leonetti@uniroma1.it (E.L.); antonio.filippini@uniroma1.it (A.F.); anna.riccioli@uniroma1.it (A.R.); 2 Department of Surgery “Pietro Valdoni”, “Sapienza” University of Rome, 00161 Rome, Italy; alessandra.cucina@uniroma1.it; 3 Azienda Policlinico Umberto I, 00161 Rome, Italy; 4 Istituto di Istologia ed Embriologia, Dipartimento di Scienze della Vita e Sanità Pubblica, Fondazione Policlinico Universitario A. Gemelli IRCCS, 00168 Rome, Italy; BiancaMaria.Scicchitano@unicatt.it (B.M.S.); silvia.sorrentino@unicatt.it (S.S.); 5 Department of Experimental Medicine, “Sapienza” University of Rome, 00161 Rome, Italy; mariagrazia.tarsitano@uniroma1.it (M.G.T.); andrea.isidori@uniroma1.it (A.I.); mariano.bizzarri@uniroma1.it (M.B.); 6 Systems Biology Group Lab, 00161 Rome, Italy; 7 Department of Experimental Medicine, Università degli Studi della Campania “Luigi Vanvitelli”, 80138 Naples, Italy; marcella.cammarota@unicampania.it; 8 Istituto Nazionale Tumori IRCCS “Fondazione G. Pascale”, 80131 Naples, Italy; gigantino.vincenzo@gmail.com

**Keywords:** TGCTs, c-MET, HGF, PI3K, PI3K inhibitors, cancer therapy

## Abstract

Overactivation of the c-MET/HGF system is a feature of many cancers. We previously reported that type II testicular germ cell tumor (TGCT) cells express the c-MET receptor, forming non-seminomatous lesions that are more positive compared with seminomatous ones. Notably, we also demonstrated that NT2D1 non-seminomatous cells (derived from an embryonal carcinoma lesion) increase their proliferation, migration, and invasion in response to HGF. Herein, we report that HGF immunoreactivity is more evident in the microenvironment of embryonal carcinoma biopsies with respect to seminomatous ones, indicating a tumor-dependent modulation of the testicular niche. PI3K/AKT is one of the signaling pathways triggered by HGF through the c-MET activation cascade. Herein, we demonstrated that phospho-AKT increases in NT2D1 cells after HGF stimulation. Moreover, we found that this pathway is involved in HGF-dependent NT2D1 cell proliferation, migration, and invasion, since the co-administration of the PI3K inhibitor LY294002 together with HGF abrogates these responses. Notably, the inhibition of endogenous PI3K affects collective cell migration but does not influence proliferation or chemotactic activity. Surprisingly, LY294002 administered without the co-administration of HGF increases cell invasion at levels comparable to the HGF-administered samples. This paradoxical result highlights the role of the testicular microenvironment in the modulation of cellular responses and stimulates the study of the testicular secretome in cancer lesions.

## 1. Introduction

Testicular germ cell tumors (TGCTs) are a group of neoplasms whose incidence is continuously increasing and mostly affects young men. These cancers have an excellent prognosis, however, without adjuvant treatment, approximately 30% of non-seminomatous germ cell tumors (NSGCT) and 15% of seminomatous lesions relapse [1]. Platinum-based chemotherapy has improved the survival rate up to 90%, even though a percentage of patients still develop chemo-resistance, and a subset of them die due to disease progression [2,3]. Moreover, it is fair to highlight that adjuvant chemotherapy is associated with detrimental platinum-associated side effects [4,5], and this evidence is particularly relevant considering the young age of the patients at the onset of the disease. For these reasons, in the last decade, there have been efforts to find alternative therapeutic strategies [3] in order to achieve the goal of further improving the clinical outcome and, at the same time, minimizing therapy-related toxicity.

The investigation of the molecular features of TGCTs would help in identifying novel therapeutic targets that in turn could improve treatment outcomes, contributing to the development of a more personalized therapy. However, the exploration of possible second-generation therapies in pre-clinical studies is still limited [3,6].

Tyrosine-kinase receptors and associated signaling pathways are often involved with the onset and progression of many types of cancerous lesions. Among them, the c-MET/HGF-activated pathway is one of the most studied: its deregulation or constitutive activation is associated with cancer onset and progression, and for this reason, it has been considered as a potential target for therapeutic purpose in several solid cancers [7].

Our group demonstrated, for the first time, that the c-MET receptor is expressed in biopsies derived from patients affected by TGCTs (both seminomatous and non-seminomatous lesions, but at a higher level in the non-seminomatous lesions with respect to the seminomatous ones [8]). Notably, in the same paper, we demonstrated that non-seminoma-derived NT2D1 cells respond to HGF administration, increasing their proliferative and migratory index.

We also demonstrated that c-Src inhibition abrogates the HGF-dependent increase of cell proliferation, polarized and collective migration, as well as cell invasion [9]. In the same paper, we found that, in basal culture conditions, c-Src inhibition decreases the cell proliferation rate of NT2D1 cells, independently from c-MET pathway activation, indicating that c-Src is used by other constitutively activated pathways that are responsible of activation of the cell cycle. Notably, we also found that c-Src inhibition, when administered in basal culture conditions, increases NT2D1 invasiveness via a HGF-independent way, highlighting the importance of the microenvironmental cues in modulating cellular responses to pharmacological stimuli [9]. All together, these observations led us to further investigate the c-MET-triggered signal transduction pathway in non-seminoma cell malignant behavior.

In this paper, we focused on c-MET-activated PI3K/AKT signaling, since its role in the progression of several solid oncological lesions is well known [10,11,12]. PI3Ks are members of a conserved family of lipid kinases which are grouped into three classes. The members of class I are the most studied in cancer physiology [13,14]. Notably, systemic inhibition of p110β PI3K using a knock-in mouse model showed blockade maturation of spermatogonia that fail to enter meiosis [15]. It has been reported that PTEN prevents PI3K/AKT activation, and intriguingly, PTEN downregulation has been reported during the transition from germ cell neoplasia in situ (GCNIS) to invasive TGCTs [16]. It is well known that PI3K is often recruited together with c-Src after c-MET activation [17,18], and notably, the constitutive activation of both PI3K and/or c-MET is considered to be involved in the acquisition of chemo- and radio-resistance of oncological lesions [19,20,21,22,23,24]. In the present study, we investigated the role of PI3K in the HGF-dependent and c-MET-activated malignant behavior of NT2D1 non-seminoma cells, studying the effects of PI3K inhibition on the already described biological responses to HGF (proliferation, migration, and invasion).

## 2. Results

### 2.1. HGF Distribution Pattern in TGCT Histological Samples

In a previous work, we evaluated c-MET expression in histological samples derived from all type II TGCTs. In that paper, seminomatous lesions were scored with a lower c-MET membranous staining with respect to the non-seminomatous ones [8].

**Figure 1:**
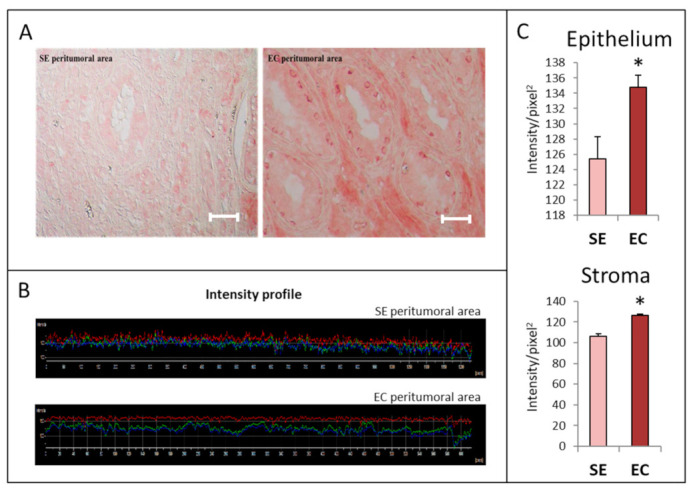

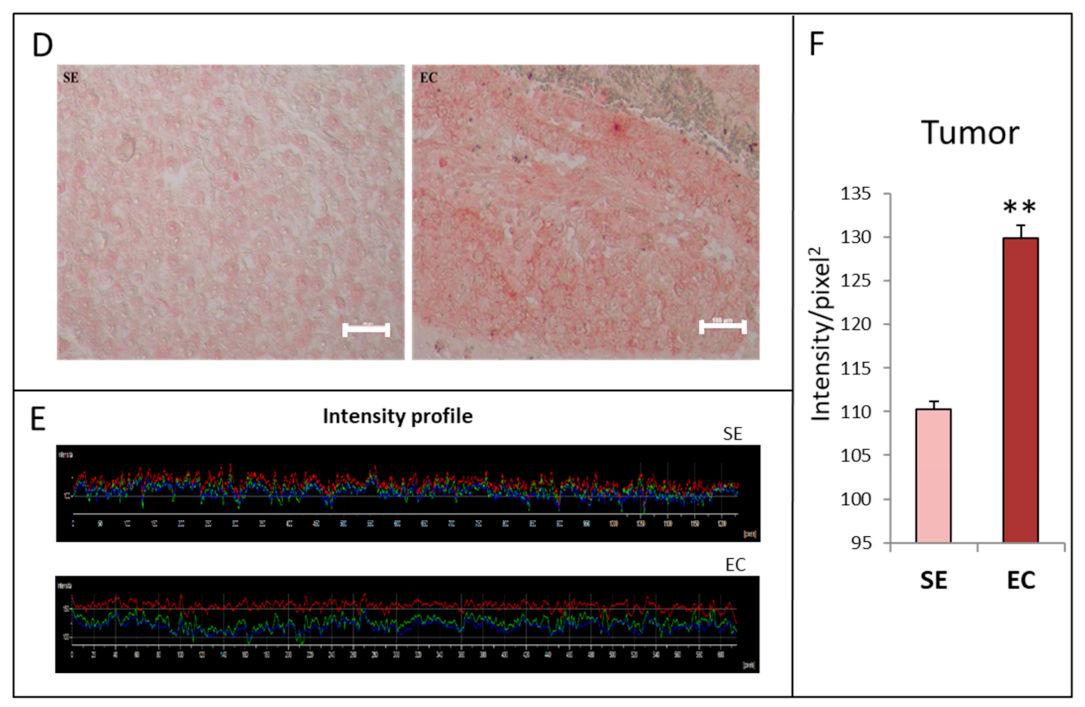
Representative images of HGF immunoreactivity (**A**,**D**) in seminoma (SE) and embryonal carcinoma (EC) samples (**D**), and their peritumoral areas (**A**). Representative intensity profiles of immunohistochemical experiments are shown in panel (**B**) (peritumoral areas) and (**E**) tumoral lesions. (**C**) Graphical representation of the quantification of HGF immunostaining in epithelial and stromal parts of SE and EC peritumoral areas. (**F**) Graphical representation of the quantification of HGF immunostaining in SE and EC samples. Four different SE histological samples and two EC histological samples were examined. Bar: 100 µm. * *p* < 0.005; ** *p* < 0.001.

Herein, we report the HGF immunoreactivity of samples from patients affected by seminoma (SE, four patients) or embryonal carcinoma (EC, two patients). We decided to analyze EC samples since NT2D1 cells originate from an embryonal carcinoma lesion. Moreover, EC is at the crossroad of all non-seminomatous lesions. We observed that HGF expression is higher in EC with respect to SE samples (Figure 1D–F). Notably, HGF appears to mainly be localized in the cytoplasm of SE cells, whereas it appears more widely diffused in EC lesions (Figure 1D). Intriguingly, we observed that the EC peritumoral area also demonstrates stronger immunoreactivity with respect to its SE counterpart (Figure 1A–C). Notably, atrophic tubules in the EC peritumoral areas show a featured perinuclear HGF signal in basal germ cells (Figure 1A).

### 2.2. The PI3K/AKT Pathway Is Activated after HGF Administration in NT2D1 Cells

It is well known that the HGF/c-MET system is able to activate the PI3K/AKT pathway, even though no data are available so far concerning the activation of this pathway in NT2D1 cells. We previously demonstrated that NT2D1 cells do not express and secrete HGF [8]; therefore, as far as we know, there is not an autocrine contribution to c-MET activation in this cell line. In line with this result [25,26], Selfe and coworkers studied the constitutive phosphorylation of tyrosine-kinase receptors in TGCT-derived cell lines and concluded that the c-MET receptor is not constitutively activated in NT2D1 cells.

**Figure 2:**
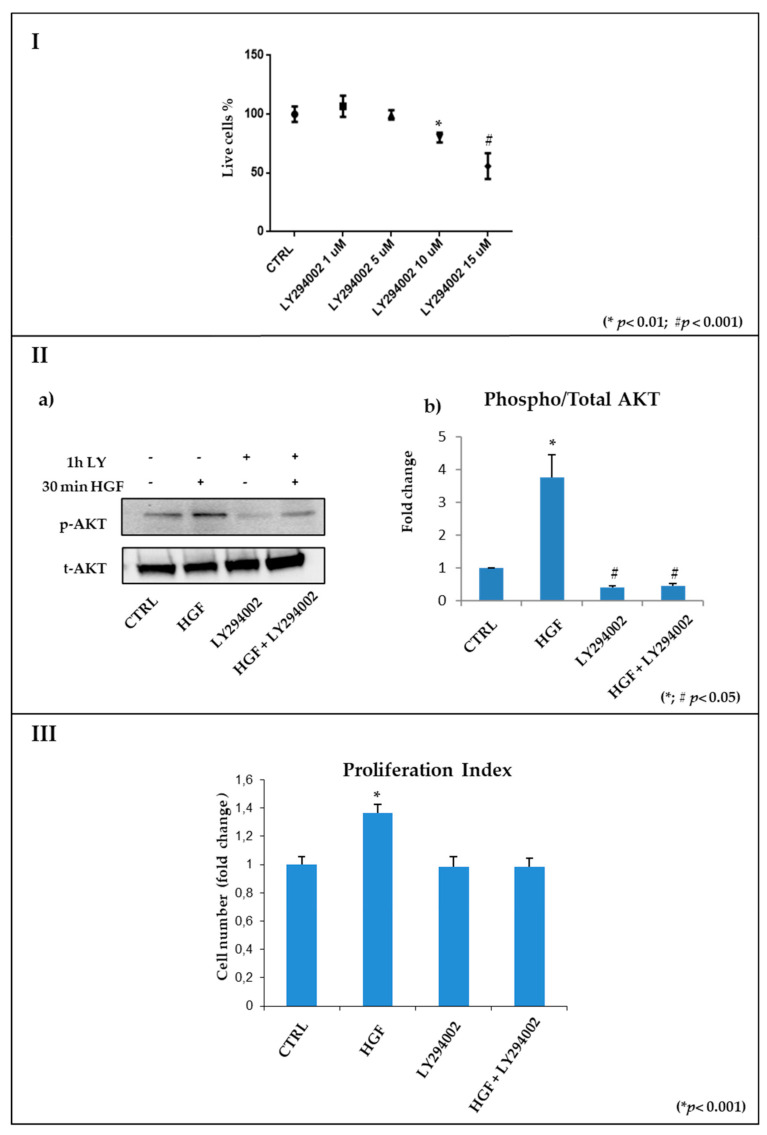
(**I**) Cell death Flow Cytometry nalysis. Graphical representation of the percentage of live cells obtained by culturing NT2D1 cells with different concentrations of LY294002 for 48 h (* *p* < 0.01; # *p* < 0.001). (**II**) Western blot analyses of p-AKT and total AKT in NT2D1 cell lines cultured in basal conditions (CTRL), with 5 µM LY294002, with 40 ng/mL HGF, and with LY294002 + HGF. On the left: representative images of p-AKT and total AKT bands, obtained by using stain-free technology (Bio-Rad Laboratories Inc., Hercules, CA, USA), are shown. On the right: the densitometric analysis of pAKT/AKT bands is reported (*; # *p* < 0.05). (**III**) Graphical representation of the number of NT2D1 cells cultured for 48 h in control conditions, with HGF, with LY294002, or their combination. Cells cultured with HGF had a high proliferative rate (* *p* < 0.001). Results were expressed in fold change, with the control considered as 1 (±standard error of the mean (SEM)).

To assess HGF-dependent PI3K/AKT pathway activation, Western blot analysis of p-AKT and total AKT has been performed on NT2D1 cells cultured for 30 min in basal conditions and after HGF administration (Figure 2, panel II). The results clearly show a significant increase in the pAKT/AKT ratio in HGF-treated samples, indicating activation of the PI3K-dependant pathway. All Western blots performed to assess AKT activation are reported in Figure S2.

### 2.3. Pharmacological Inhibition of PI3K/AKT in Culture Using LY294002

In the present paper, we pharmacologically inhibited the PI3K activity by administering the PI3K inhibitor LY294002 in culture, with or without the stimulation of HGF. We used this strategy to test the involvement of class I PI3Ks in HGF-dependent and HGF-independent NT2D1 cell proliferation, migration, and invasion.

#### 2.3.1. Identification of the Effective and Non-Toxic Concentrations of LY294002

To identify the non-toxic dose of LY294002 in NT2D1 cells, we performed cell death Flow Cytometry analysis by culturing NT2D1 cells with different concentrations of the inhibitor (1, 5, 10, 15 µM) for 48 h. These experiments demonstrated that there is no statistically significant difference in live cell percentage with respect to control conditions when the inhibitor is used at 1 and 5 µM (about 106% ± 5 for 1 µM and 99% ± 2 for 5 µM when control is reported as 100%). Starting from 10 µM, the inhibitor causes a significant decrease in cell viability compared to the control conditions (about 80% ± 2 for 10 µM and 55% ± 6 for 15 µM when control is reported as 100%) (Figure 2, panel I). A Trypan blue exclusion test was also performed and confirms these data (not shown). From these results, 5 µM LY294002 appears to be the highest dose that could be used in culture while avoiding toxic effects. At least three independent experiments were performed in triplicate.

#### 2.3.2. The PI3K/AKT Pathway Is Inhibited by LY294002 Administration in NT2D1 Cells

In order to assess the capability of 5 µM LY294002 to inhibit the PI3K/AKT pathway, Western blot analyses of p-AKT and total AKT were performed on NT2D1 cells cultured in basal conditions (CTRL), or in the presence of 5 µM LY294002, or HGF 40 ng/mL, or the combination HGF and LY294002. Densitometric analysis of the bands demonstrated that after 30 min of treatment, LY294002 affected the endogenous phosphorylation with respect to the control condition, which was considered as 1 (0.35 ± 0.06 vs. 1 ± 0.02 *p* < 0.05). HGF alone significantly increased the phosphorylation of AKT in the activator site (Ser 473) (HGF 2.15 ± 0.24 vs. CTRL 1 ± 0.02 *p* < 0.05), while 5 µM LY294002 in combination with HGF was able to revert this cellular response (0.40 ± 0.06 vs. HGF 2.15 ± 0.24 *p* < 0.05) (Figure 2, panel II). These results indicate that that the PI3K/AKT phosphorylation pathway, both endogenous and HGF-triggered, is inhibited by LY294002 administration. Three independent experiments were performed. All Western blots performed to assess AKT phosphorylation are reported in Figure S2.

### 2.4. HGF-Stimulated NT2D1 Cell Proliferation Depends on PI3K/AKT Activation

We previously demonstrated that HGF specifically determines c-MET activation in NT2D1 cells [8], leading to a significant increase in NT2D1 cell proliferation after 48 h of culture. To test whether PI3K/AKT is involved in this biological response, NT2D1 cells were cultured for 48 h as follow: basal condition (CTRL), LY294002 5 µM, HGF 40 ng/mL, or the combination of HGF and LY294002. Cell numbers were not affected by LY294002 when administered alone compared to the values with those obtained in basal conditions (0.98 ± 0.07 vs. 1 ± 0.06 respectively; *p* = n.s.). As expected, HGF induced a significant increase in cell number after 48 h of culture with respect to the control conditions (1.36 ± 0.06 vs. 1 ±.0.06 respectively; *p* < 0.001), and, notably, the combination of HGF + LY294002 completely abrogates the HGF-induced increase in cell number (the values were similar to the control condition: 0.98 ± 0.06 vs. 1 ± 0.06 respectively; *p* = n.s.). We already demonstrated that the HGF-dependent increase in cell number is due to the activation of the cell cycle and it is not due to an increase in cell survival [8,9]; therefore, we can state that PI3K activity is involved in the HGF-dependent proliferation of NT2D1 cells (Figure 2, panel III). At least three independent experiments were performed in triplicate.

### 2.5. SEM Analysis of HGF-Induced NT2D1 Morphological Modification: Effect of PI3K Inhibition

To study whether c-MET/HGF pathway activation could modify NT2D1 cell shape, membrane surface morphology and activity, cells were treated for 24 h with HGF and analyzed by scanning electron microscopy (SEM), as described in the Materials and Methods section.

**Figure 3:**
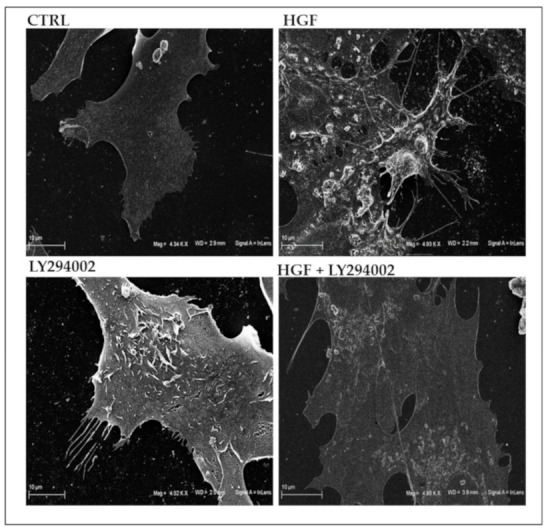
Scanning electron microscopy analysis. Representative images of NT2D1 cells cultured for 24 h in control conditions, or treated with HGF, LY294002, or their combination. Scale bar: 10 μm.

This analysis revealed that HGF-treated cells significantly modify their shape, appearing stretched, and strongly were characterized by the presence of membrane protrusions, such as microvilli-like structures or membrane ruffles. Moreover, SEM analysis demonstrates that HGF stimulation induces micro-vesicle formation on the cell surface and their deposition on the substrate of the plate. This experimental condition also determines the formation of filopodia and lamellipodia, confirming the migratory attitude induced by HGF administration in this cell line. On the contrary, control cells have a smooth membrane surface and membrane activity appears less evident (Figure 3).

We also performed SEM analysis on LY294002 treated cells with or without HGF stimulation. We observed that the co-administration of LY294002 with HGF reverts the membrane morphology to the control condition. Intriguingly, LY294002 alone also appears to affect NT2D1 cell membrane activity, inducing the appearance of filopodia and microvilli-like structures, but to a lesser extent with respect to HGF administration (Figure 3). This result was surprising but highlights the relevance of microenvironmental cues in the modulation of NT2D1 cell behavior. Moreover, it explains, at least in part, the results that we obtained in the invasion assay (see below).

### 2.6. The PI3/AKT Pathway Is Involved in HGF-Dependent NT2D1 Chemotaxis

**Figure 4:**
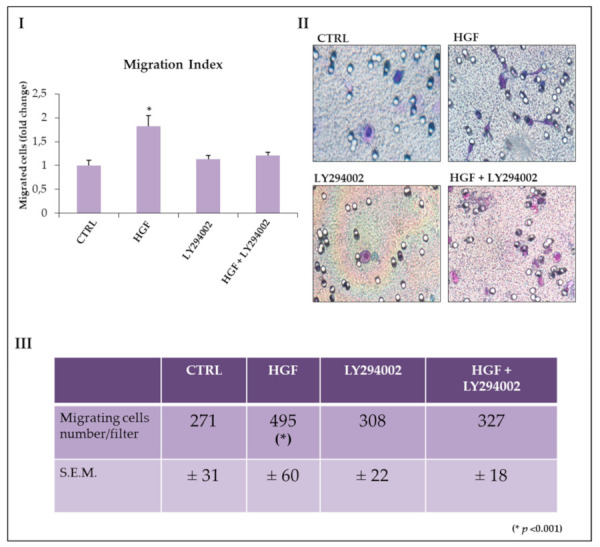
Effect of LY294002 on cell NT2D1 cell migration. (**I**) Quantitative analysis of chemoattracted NT2D1 cells. The values were calculated as “fold change” (±S.E.M.) compared to the control, which was considered as 1. The use of LY294002 in combination with HGF abrogates the migratory effect induced by HGF. (**II**) Representative images of NT2D1 cell migration. Images were recorded at 40× magnification. (**III**) Table illustrating the number of migrating cells/filter (* *p* < 0.001). At least three independent experiments were performed in triplicate.

We established that HGF acts as a chemo-attractant for NT2D1 in Boyden chamber migration assays [8]. We also demonstrated that this migration is c-MET specific and that c-Src recruitment is involved in this process [9]. In this work, we analyzed the possible involvement of the PI3K/AKT pathway in HGF-induced chemotaxis. We performed migration experiments using the already mentioned PI3K inhibitor, LY294002. The inhibitor alone did not affect migration rate with respect to the control condition (1.1 ± 0.08 vs. 1 ± 0.1 respectively; *p* = n.s.). As shown in Figure 4, as expected, cell migration significantly increased in the presence of HGF with respect to the control (1.8 ± 0.2 vs. 1 ± 0.1 respectively; *p* < 0.001). The co-administration of HGF and LY294002 significantly reduces HGF chemoattraction in NT2D1 cells (1.2 ± 0.07 vs. 1.8 ± 0.2 respectively; *p* < 0.001), reverting cell migration to control values. Taken together, these results confirm that NT2D1 chemotaxis is HGF-dependent and demonstrate that the PI3K/AKT pathway is involved in this phenomenon.

### 2.7. The PI3K/AKT Pathway Is Involved in HGF-Dependent and HGF-Independent NT2D1 Cell Invasion

**Figure 5:**
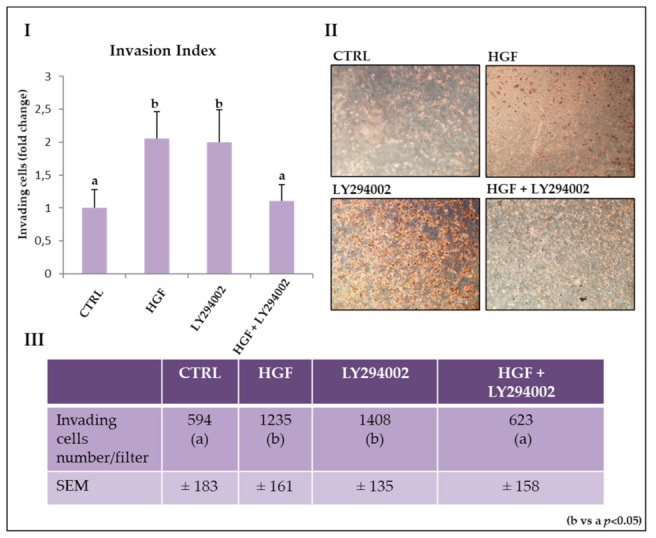
Effect of LY294002 on NT2D1 cell invasion. (**I**) Quantitative analysis of invading cells. Results are expressed as fold change (±S.E.M.) and the control condition is considered as 1 (b vs. a; *p* < 0.05). (**II**) Representative phase contrast images of invading cells under different culture conditions. Images were recovered at 10× magnification. (**III**) Table illustrating the number of invading cells/filter in all experimental conditions. At least three independent experiments were performed in triplicate.

In our previous works, we demonstrated that HGF stimulates NT2D1 cell invasion. We also demonstrated that c-Src is involved in HGF-dependent and independent modulation of NT2D1 cell invading behavior [8,9]. To better describe this phenomenon, we performed invasion assays inhibiting PI3K activity with LY294002. The results obtained are summarized in Figure 5, and confirm our previous data, demonstrating that HGF administration significantly increases invading cell number with respect to the control condition (2.0 ± 0.27 vs. 1 ± 0.3 respectively; *p* < 0.05). When LY294002 was administered in combination with HGF, the number of invading cells reverted to the control values (1.05 ± 0.26 vs. 1 ± 0.3 respectively; *p* = n.s.). Surprisingly, we observed that LY294002 administered alone significantly increases NT2D1 cell invasion with respect to the control condition (2.37 ± 0.2 vs. 1 + 0.3 respectively; *p* < 0.05), resulting in values similar to those obtained after HGF administration (2.0 ± 0.27 vs. 2.37 ± 0.2; *p* = n.s.) (Figure 5). These results demonstrate a crucial role for the PI3K/AKT pathway in HGF-dependent NT2D1 cell invasion, but also highlight that the inhibition of PI3K, in the absence of HGF stimulation, has a paradoxical effect on NT2D1 cell invasion.

This phenomenon is remarkably comparable to what we observed using SRC inhibitor-1 [9] and it highlights the relationship between PI3K and c-Src activated pathways. A possible explanation for these results is that the endogenous ability of NT2D1 cells to invade the extracellular matrix is microenvironment-dependent, and therefore the presence or absence of HGF is a crucial factor for the different biological responses seen in the NT2D1 cell line. In the light of these results, the observations obtained by SEM analyses (in which we observed a significant increase in membrane protrusions in NT2D1 cells after HGF and LY294002 were singly administered) make sense and let us speculate that cytoskeleton remodeling occurred in the presence of LY294002, even when administered alone.

### 2.8. The PI3K/AKT Pathway Is Involved in the Modulation of Both Constitutive and HGF-Induced Collective Migration

HGF-dependent c-MET activation is able to induce collective migration in NT2D1 cells, as we previously demonstrated [8]. To investigate the involvement of PI3K in this phenomenon, we performed a wound healing assay in the previously described culture conditions.

**Figure 6:**
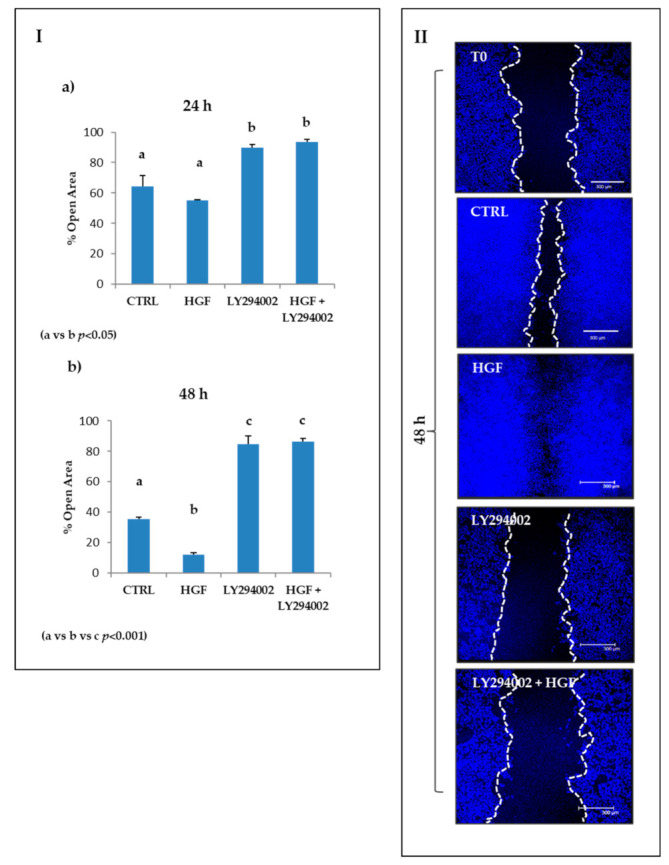
Effect of LY294002 on NT2D1 cell collective migration. (**I**) Quantitative analysis of wound closure after 24 h (**a**) and 48 h (**b**). Data are expressed as the mean percentage of residual open area compared with the respective T0 condition. At 24 h, the decrease of open area in HGF-treated cells was not statistically significant compared with the control condition, but the closure was almost complete at 48 h (a vs. b, *p* < 0.001). LY294002 in combination with HGF at 24 h (b vs. a, *p* < 0.01) and 48 h (c vs. b, *p* < 0.001) abrogated the migratory effect induced by HGF. LY294002 alone was also able to inhibit the collective migration of the cells when cultured for 24 h (b vs. a, *p* < 0.05) and 48 h (c vs. a, *p* < 0.001). (**II**) Representative images of nuclei in the wound healing assay, recovered immediately after insert removal (T0) and 48 h after wounding. Images were photographed at 10× magnification (scale bar: 300 µm). At least three independent experiments were performed in triplicate.

As expected, HGF administration caused an evident collective cell migration that was inhibited in the presence of the PI3K inhibitor, both at 24 h and at 48 h of treatment (Figure 6). In more detail, after 24 h of culture, the open residual area of samples treated with LY294002 alone or LY294002 in combination with HGF showed values similar to its own T0 result (90% ± 2.1 and 93.4 ± 1.7 respectively) and, therefore, were significantly higher compared with control conditions (64% ± 7; *p* < 0.05) and HGF-treated wells (55% ± 0.6 *p* < 0.05) (Figure 6, Panel I a). This difference was even more significant after 48 h of culture: the value of the open residual area of samples treated with LY294002 alone (84% ± 5) or LY294002 in combination with HGF (86% ± 2) remained almost unchanged with respect to its own 24 h result (*p* = n.s.), but was significantly higher compared with the control condition (35.4% ± 1.5) or HGF-treated samples (12.2% ± 1) (*p* < 0.001) (Figure 6 Panel I b; Panel II). Notably, the co-administration of HGF + LY294002 blocks the collective migration of NT2D1 cells at levels even below the basal culture condition.

These data demonstrate the involvement of PI3K in both the constitutive and HGF-induced collective migration of NT2D1 cells. Even in this case, the results obtained are remarkably similar to those we already published using Src inhibitor-1 [9], highlighting again the tight relationship between c-Src and PI3K activation in NT2D1 cells.

### 2.9. Cytoskeletal Remodeling Is Involved in HGF-Induced Collective Migration: The Role of PI3K

**Figure 7:**
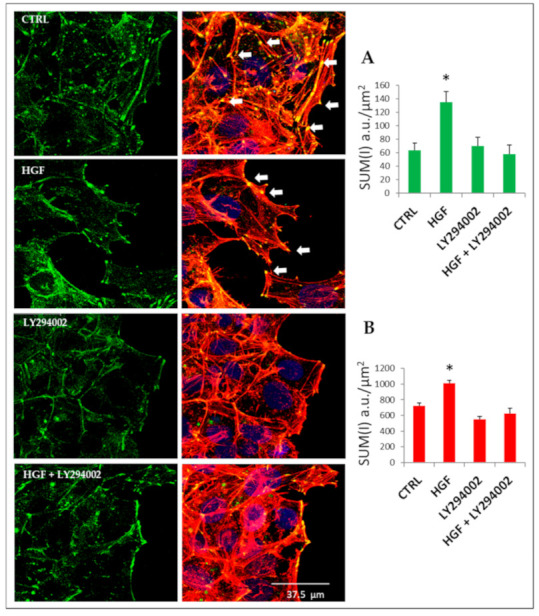
Left panel: representative double-fluorescence confocal images of vinculin immunostaining (green signal) and F-actin (red signal) to identify focal adhesion (FA) organization in NT2D1 cells during the wound healing experiment. Left: vinculin immunostaining (green signal); right: merged picture of vinculin with F-actin (red signal). White arrows indicate FAs. Scale bar: 37.5 µm. Right panel: (**A**) quantitative analysis of total vinculin carried out by Leica confocal software SUM(I). (**B**) Quantitative analysis of total F-actin carried out by Leica confocal software SUM(I). (* *p* < 0.05); a.u. = arbitrary units.

To better investigate cell morphology and cytoskeleton reorganization during NT2D1 collective migration, both in basal conditions and after HGF administration, confocal analysis of the F-actin distribution pattern at the migration front of the scratch region using TRITC-conjugated phalloidin was performed. As expected, in HGF-treated cells at 48 h post-scratch, the wound area was almost completely closed with respect to the control condition (Figure S1). In both in the control and in HGF-treated cells, confocal analysis showed the organization of cytoskeletal actin in peripheral cortical bundles and stress fibers (Figure 7), which are features of collective migration capability [27].

At the migration front of HGF-exposed cells, in addition to the cortical bundles, we also observed lamellipodia and membrane ruffles more frequently with respect to the control (Figure S1 dashed line). Interestingly, when NT2D1 cells were treated with LY294002 alone or in combination with HGF, stress fibers decreased drastically, together with detectable lamellipodia (Figure 7). The quantitative evaluation of F-actin using confocal software (Sum of intensity: (SUM(I) indicates an increase in F-actin after HGF administration, and that LY294002 co-administration completely reverts this phenomenon. These observations are in line with the reported inhibition of collective migration exerted by PI3K inhibition (Figure 6).

### 2.10. HGF Induces Focal Adhesion Formation during Collective Migration via PI3K Stimulation

To clarify the adhesive/cytoskeletal modifications that occur at the leading edge of NT2D1 migrating cells, we studied the distribution pattern of vinculin in the wound healing samples after 24 h of culture (Figure 7). This timepoint was chosen because at 24 h, the wound is not completely closed, and therefore the morphology of the cell leading edge is observable.

**Figure 8:**
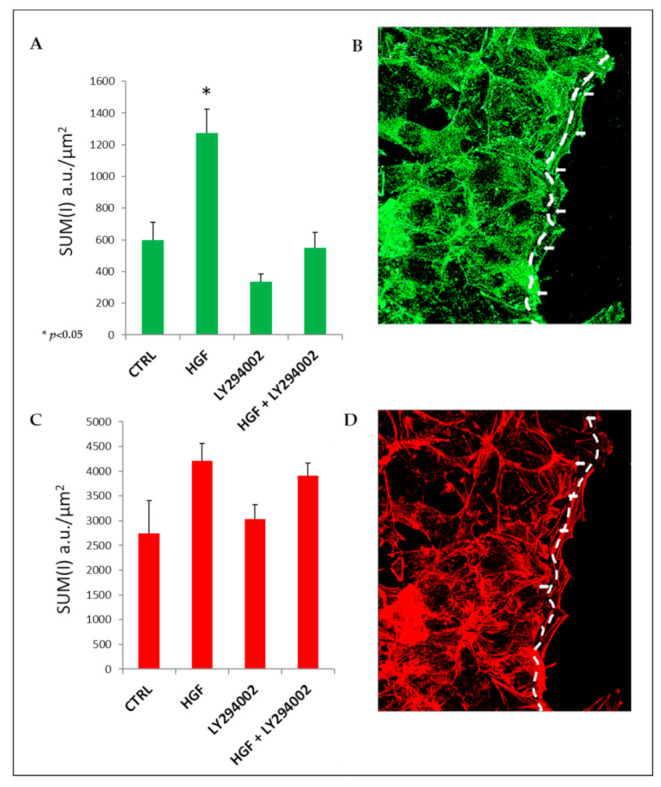
(**A**) Quantitative analysis of vinculin at the leading edge of cell migration carried out by confocal microscopy. (* *p* < 0.05) (**B**) Representative image of vinculin immunofluorescence (green), in which the region that has been considered for vinculin quantification has been highlighted (dashed lines; (SUM(I)/µm^2^). (**C**) Quantitative analysis of F-actin at the leading edge of cell migration carried out by confocal microscopy. (**D**) Representative image of F-actin staining (red) in the highlighted region (dashed lines) has been considered for F-actin quantification (SUM(I)/µm^2^).

Vinculin is a key element that is necessary for mechanical signaling, and when it binds to F-actin, it is critical for cell migration. Moreover, it is present in focal adhesions (FAs) [28]. Vinculin-featured FAs are clearly observable in both control and HGF-treated samples (Figure 7, arrows), but they are more prominent in samples in which more F-actin stress fibers are found. The quantitative analysis of total vinculin reflects the F-actin quantification profile (Figure 7A,B). Moreover, the quantitative analysis of vinculin, carried out by confocal microscopy at the leading edge of wound healing experiments, showed that HGF triggered a significant increase in vinculin at the migration front of the scratch region with respect to control conditions (1213.33 ± 124.73 vs. 600.73 ± 110.50 respectively; *p* < 0.05). Notably, HGF + LY294002 administration significantly reverted the HGF-mediated vinculin increase (550 ± 99.41 vs. 1213.33 ± 124.73 *p* < 0.05) to values similar to the control (600.73 ± 110.50). Interestingly, the mean values of vinculin obtained in the samples treated with LY294002 alone are lower with respect to the control (335 ± 50 vs. 600 ± 110.50), even if the difference after 24 h of treatment is not statistically significant (Figure 8).

## 3. Discussion

In the last few decades, it has been increasingly highlighted in scientific literature that cancer promotion and survival depend on the complex signaling network between tumor cells and the surrounding microenvironment [29]. This tight relationship is particularly studied in TGCTs, for which the word “genvironment” has been coined, which designates the close interaction between environmental factors, diffusible signals, and gene expression regulation in the onset and progression of TGCTs [30,31,32]. Notably, these tumors are notable for low rates of somatic mutations, which is exceptional for solid cancers in adults [33]. A previous paper from our group demonstrated that the c-MET receptor is expressed in tissue biopsies derived from both seminomatous and non-seminomatous lesions, but non-seminoma-derived biopsies had a significantly higher expression of this receptor on the cell membrane [8]. In line with this observation, in the same paper, we also found that NT2D1 cells, derived from embryonal carcinoma (the lesion from which all non-seminomas originate), increase their malignant behavior in response to HGF administration, whereas TCam-2 seminoma cells do not respond to HGF, at least for the biological responses taken into account in that study.

In the light of these results, we wondered whether there was a difference in testicular HGF availability in seminoma and embryonal carcinoma biopsies. It is well known that HGF is present in the testis and male reproductive tract [34,35,36], and it was reported that the level of circulating HGF and other cytokines is inversely correlated with the progression-free survival of TGCT patients [37]. Immunohistochemical analysis of HGF reported herein clearly indicated that embryonal carcinoma samples have stronger immunoreactivity to HGF with respect to seminoma samples. Moreover, HGF immunoreactivity in seminoma samples is mainly localized to the cytosol of the cells, whereas in embryonal carcinoma, HGF appears to be distributed mainly outside the cells. This observation is relevant and sheds new light on the importance of the testicular niche of TGCT patients and depicts that testicular cells from patients with embryonal carcinoma are exposed to higher levels of HGF with respect to cells from patients with seminoma. Notably, c-MET availability and activation has been related to resistance to radio- and chemotherapy in different cancer types [7,22,38,39]; therefore, it is conceivable to hypothesize that c-MET receptor activation could lead to refractory disease in embryonal carcinoma as well. The results reported herein stimulate further investigations to evaluate this hypothesis.

Even if the embryonal carcinoma cells from patients are positive for HGF, NT2D1 cells do not secrete or express this factor [8], and this observation again highlights the importance of the microenvironment in the modulation of embryonal carcinoma cellular physiology. To study the pathway triggered by HGF on embryonal carcinoma cells, we stimulated NT2D1 cells in vitro with HGF and inhibited specific pharmacological adaptor proteins. In this study, we expanded on previous work [9] in which we studied the role of c-Src in the HGF-dependent and c-MET-activated signaling pathway, focusing our attention on another important component of the c-MET pathway: PI3K/AKT. It is fair to highlight that the PI3K/AKT pathway is often overactivated in cancer progression and that c-Src and PI3K can be recruited together after c-MET activation [40,41,42].

In our experimental model, PI3K inhibition abrogates the HGF-dependent increase of cell proliferation, polarized and collective migration, and cell invasion. The analysis of the F-actin distribution pattern and quantification revealed that PI3K is essential for stress fiber formation; this result is in line with previous papers in which stress fiber formation was notably reported as a key element for cell migration [27,43,44,45]. The amount of vinculin at the leading edge of collectively-migrating cells follows the migratory attitude observed in the differently treated samples, and explains the capability of LY294002 to revert the increase of vinculin positivity observed in HGF-treated samples in the wound-healing assay.

Interestingly, the administration of LY294002 alone does not affect HGF-independent NT2D1 cell proliferation, or cell migration in chemotaxis assays, indicating that this adaptor is specifically recruited by c-MET to trigger HGF-mediated cellular responses. However, the administration of LY294002 in basal culture conditions increases NT2D1 invasiveness and, at the same time as previously mentioned, decreases the collective migration capability of NT2D1 cells independently from c-MET pathway activation. These apparently contrasting results let us speculate that PI3K counteracts spontaneous epithelium–mesenchyme transition (EMT) in basal conditions (which is a feature of invading cells), but it changes its role when it is recruited by specific EMT cues (such as HGF), promoting cell invading behavior and, as a consequence, EMT. These results are reinforced by SEM analysis in which LY294002, when administered alone, triggers filopodia formation in NT2D1 cells. On the other hand, it should be highlighted that our results clearly indicate that constitutive PI3K activation is necessary for spontaneous collective migration in which EMT does not occur.

It is worth mentioning that these paradoxical results are remarkably comparable to what wa obtained in a previous paper by inhibiting c-Src, which allows us to speculate that both c-Src and PI3K are related proteins that are probably recruited by housekeeping homeostatic pathways to modulate the aggressive behavior of NT2D1 cells [25]. In this regard, it is fair to highlight that Selfe and co-workers [25] revealed a panel of tyrosine-kinase receptors that are constitutively phosphorylated in NT2D1 cells and act as possible activators of both c-Src and PI3K (such as IGFR, FGFR, PDGFR, and VEGFR). Moreover, downregulation and/or loss of function mutations of PTEN [16] and Spry4 [46,47] were found in TGCTs and NT2D1 cells, and both genes are involved in the prevention of AKT activity. PIK3CA and AKT1 mutations have been identified in cisplatin-resistant germ cell tumors [6,48], and phospho-AKT levels are significantly higher in cisplatin-resistant TGCTs compared with cisplatin-sensitive ones [49]. These observations, together with the results reported herein on proliferation and migration, allow us to speculate that the inhibition of AKT phosphorylation could induce the re-sensitization of tumor cells. Selfe and co-workers [25] already proposed AKT as a target for personalized therapies in TGCT refractory disease. However, the reported results of the NT2D1 cell invasion assays strongly indicate that the autologous microenvironment could positively or negatively modulate the cellular response to pathway inhibitors, and should be studied as a fundamental co-factor necessary for the success of inhibitor-based therapies. Taken together, these results indicate that the study of the testicular secretome of TGCT patients could be a useful tool to identify the possible interaction among PI3K inhibitors, i.e., those used for the targeted therapy proposed by [26] Selfe and coworkers, and the signaling pathways simultaneously present in the testicular niche that can modulate the effect of the inhibitors, leading to paradoxical effects. This investigation would help to better understand the interaction among pathway inhibitors and the cancer microenvironment and could allow for the prediction of responders and not-responders to TGTC personalized targeted therapies.

## 4. Material and Methods

### 4.1. Immunohistochemical Analyses

Slides were prepared from biopsies of patients affected by type II TGCTs. The local review board approved the protocol for the patients, which was conducted in accordance with the Declaration of Helsinki, and patients provided written informed consent. Patients were screened for the ALCeP trial (Clinical Trials Identifier: NCT01206270; 25 September 2018). Samples were fixed in formalin and embedded in paraffin. Sections were dewaxed with toluene, hydrated with a decreasing scale of alcohols, and rinsed with distilled water (dH_2_O) and PBS without Ca++ and Mg++. Endogenous peroxidases were blocked using Hydrogen Peroxide Block (KIT Abcam, cat. ab236467) for 10 min at room temperature. After two washes in 1× PBS, sections were subjected to antigen retrieval with Tris-EDTA buffer (10 mM Tris base, 1 mM EDTA solution, 0.05% Tween 20, pH 9.0) for 10 min at high temperature. To avoid possible background staining, samples were treated with Protein Block (KIT Abcam, cat. ab236467) for 30 min. The anti-HGF primary antibody (Abcam, cat. ab83760, 1:60 dilution) was incubated overnight at 4 °C. After washes, goat anti-rabbit HRP conjugate was used for 1 h at room temperature. Then, after three washes with 1× PBS, AEC Single solution (KIT Abcam, cat. ab236467) or 3,3′-diaminobenzidine (Dako, cod. K3468) was used. Nuclei were stained with hematoxylin solution. Samples were analyzed by optical microscopy using a Nikon Eclipse. Negative controls were processed in the absence of the primary antibody and pre-immune isotype rabbit immunoglobulins (1:1000 dilution). Quantitative analysis of staining intensity was performed using the Nikon Imaging Analytical Software (NIS-Elements Analysis D 4.40.00, 64 bit).

### 4.2. Cell Culture

NT2D1 embryonal carcinoma cells were purchased from ATCC in 2015. This cell line was cultured in DMEM (Sigma Aldrich, cat. D6546, St. Louis, MO, USA) supplemented with 10% fetal bovine serum (FBS; Gibco, cat. 10270, Gland Island, NY, USA), L-glutamine (Sigma Aldrich, cat. G7513, St. Louis, MO, USA) and penicillin/streptomycin (Sigma-Aldrich, cat. P0781, St. Louis, MO, USA). After 24 h, the cells were starved for 16 h under serum-free conditions and cultured with 2% FBS. The cells were used from passage 15 to 35. To investigate the PI3K/AKT pathway, cells prepared as above were pre-treated for 1 h with LY294002 (5 µM). This inhibitor was chosen as it inhibits all class I PI3Ks, which are the most relevant for cell physiology [50]. Mycoplasma testing was routinely done with the N-GARDE Mycoplasma PCR Reagent set (Euro Clone, cat. EMK090020, Milano, Italy). The cells were treated with 40 ng/mL of human recombinant HGF (R&D Systems, cat. 294-HG), and with LY294002 (Cayman Chemical, cat.70920, Ann Arbor, MI, USA). We tested different concentrations (1, 5, 10, 15 µM) of the PI3K inhibitor, and found that 5µM was the highest concentration without toxic effects as evaluated by cell death FACS analysis and Trypan blue exclusion tests.

### 4.3. Cell Death Analysis

Cells, cultured as above, were treated with LY294002 at different concentrations (1, 5, 10, 15 µM) for 48 h. Cell death was evaluated by flow cytometry using propidium iodide (PI) exclusion assay: 2 µg/mL of PI solution (Sigma-Aldrich, cat. P4864, St. Louis, MO, USA) was added to each sample and PI fluorescence was determined by FACS (CyAn ADP, Beckman Coulter, Fullerton, CA, USA). Data were analyzed with the FCS Express 5.1 software (De Novo, Los Angeles, CA, USA).

Trypan blue exclusion tests were also used. Cells were centrifuged at 100× *g* for 5 min and the pellet was suspended in PBS. One part 0.4% Trypan blue (T6146, Sigma Aldrich, St. Louis, MO, USA) and one part cell suspension were mixed and incubated for approximately 3 min at room temperature. Then, cells with a clear cytoplasm (viable cells) and cells with a blue cytoplasm (non-viable cells) were counted within 3 min with hemocytometer and the percentage of viable cells/total number of cells was calculated.

### 4.4. Cell Proliferation Assay

For the proliferation assays, NT2D1 cells (9 × 10^4^) were cultured in 12-well plates as described above. The cells were maintained for 48 h in the presence of 2% FBS with DMEM alone (control conditions), or with LY294002 5 µM, HGF 40 ng/mL, or LY294002 + HGF. After 48 h, cells were trypsinized, harvested and counted. Each experiment was performed at least in triplicate. Three independent experiments were performed. The results (mean ± S.E.M) are expressed in fold change with the control condition considered as 1.

### 4.5. Chemotaxis Assay

Chemotaxis assays were performed using cell culture inserts (12-well, 8.0 µm pore size; Falcon, cat.353182, Lincon Park, NJ, USA) placed in a 12-multiwell (Transwell Falcon, cat. 351143, Lincon Park, NJ, USA). TCam-2 seminoma cells were used as a negative control [8]. Cells pre-treated with the inhibitor for 1 h were trypsnized, counted and resuspended in DMEM without serum. Then, 2 × 10^5^ cells/well in 1.4 mL DMEM were added in the upper chamber of the transwell in the absence (DMEM alone) or in presence of LY294002, whereas the lower chambers were filled with 800 µL DMEM (control condition) or DMEM + HGF as chemoattractants. Cells were incubated at 37 °C with 5% CO_2_. After 5 h, the medium and unmigrated cells in the upper surface of the insert were mechanically removed and the insert (containing the migrated cells in the lower surface), was fixed with 4% paraformaldehyde in PBS (pH 7.4) at 4 °C and stained with Diff Quick solution (DADE, cat. 130832, Network, NJ, USA). Migrated cells were counted under a 40× objective using an optical microscope (Axioplan Zeiss, Oberköchen, Germany) and the average number ± SEM of cells were reported as fold change with respect to the control, which was considered as 1. The whole area of each filter was counted. Three independent experiment were performed; each experiment was performed in quadruplicate at least.

### 4.6. Matrigel Invasion Assay

In vitro invasion assays were performed using chambers coated with GFR Matrigel (Basement Membrane Matrix Growth Factor Reduced; BD Biosciences, cat. 354483, San Jose, CA, USA) as previously described [9]. TCam-2 seminoma cells were used as a negative control [8]. Briefly, cells pre-treated with LY294002, were trypsinized, counted and resuspended in DMEM with 2% FBS. Then, 2.5 × 10^4^ cells/well were seeded on the top of the GFR Matrigel in 500 µL of medium alone (control condition) or containing LY294002, HGF, or both factors; the lower chambers were filled with 750 µL DMEM with 2% FBS. The cells were incubated for 24 h at 37 °C with 5% CO_2_ and then GFR Matrigel and non-invading cells were mechanically removed with a cotton swab. The polycarbonate filter containing the invading cells was fixed with 4% paraformaldehyde in PBS (pH 7.4) at 4 °C and stained with Diff Quick solution. The filter was analyzed by optical microscopy and four fields/filter were recovered at 10× magnification. Invading cells were counted and the average number ± SEM of cells were reported as fold change respect to the control, which was considered as 1. Three independent experiments were performed; each experiment was performed in triplicate at least.

### 4.7. Wound-Healing Assay (Collective Migration Assay)

Wound-healing assays were performed using special double well culture inserts (Ibidi GmbH, Martinsried, Germany). Each insert was placed in a 24-well plate and 3.5 × 10^4^ cells were placed into both wells of each insert with 70 μL of medium containing 2% FBS. At confluence, the culture inserts were gently removed, and cells were fed with fresh DMEM with 2% FBS or DMEM with 2% FBS containing HGF (40 ng/mL), LY294002 (5 µM), or both factors. Each well was photographed at 10× magnification immediately after insert removal for baseline wound measurement (T0), and after 24 h and 48 h with a Nikon DS-Fi1 camera (Nikon Corporation, Tokyo, Japan) coupled with a Zeiss Axiovert optical microscope (Zeiss, Oberkochen, Germany). TO-PRO3 iodide fluorescent dye 642/661 (1:5000 in PBS, Invitrogen, cat. T3605, Carlsbad, CA, USA) was used for nuclei staining. The mean percentage of residual open area compared with the respective open area recovered at T0 was calculated using ImageJ v 1.47 h software. For each experimental condition, four independent experiments were performed in triplicate.

### 4.8. Confocal Analysis of F-Actin and Vinculin Distribution Pattern

To describe the distribution pattern of vinculin and actin, immunofluorescence experiments were performed. Cells prepared for the wound healing assays were fixed at 24 h and 48 h in 4% paraformaldehyde in PBS (pH 7.4) at 4 °C for 15 min, and permeabilized in PBS supplemented with 1% BSA and 0.1% Triton for 2 h. Samples were then incubated overnight with mouse anti-vinculin primary antibody (Santa Cruz, cat. sc-73614, Santa Cruz, CA, USA, 1:50 dilution). Then, samples were washed three times in PBS/BSA/Triton for 30 min, and incubated with the appropriate secondary antibody: FITC-conjugated donkey anti-rabbit IgG (Jackson Immuno Research, cat. 711-095-152, West Grove, PA, USA, dil. 1:200), TO-PRO3 iodide fluorescent dye 642/661 (1:5000 in PBS, Invitrogen, cat. T3605, Carlsbad, CA, USA) for nuclei staining, and rhodamine phalloidin (Invitrogen Molecular Probes Eugene 1:40 dilution) for F-actin visualization were used. As a negative control, the primary antibody was omitted. Immunofluorescence experiments were analyzed using a Leica confocal microscope (Laser Scanning TCS SP2 equipped with Kr/Ar and He/Ne lasers, Mannheim, Germany). Laser lines were 488, 543 and 633 nm for FITC, TRITC and TO-PRO3 excitation, respectively. The images were scanned under a 20× or 40× oil immersion objective. Co-localization analysis (FITC/green signal and TRITC/red signal) was performed by Leica confocal software, SUM(I). To perform quantitative analysis of fluorescence, optical spatial series with a step size of 1 µm were recovered. The fluorescence intensity of vinculin and F-actin was determined by maximum projection in sized regions of interest (ROI) drawn on the whole field of each series, or at about 6 µm from the migration front using Leica confocal software. Three independent experiments in duplicate were analyzed.

### 4.9. Western Blot Analyses

To investigate the PI3K/AKT pathway, cells prepared as described above were cultured with DMEM alone or HGF, LY294002, or HGF + LY294002 for 30 min. Then, cells were solubilized in lysis buffer (1% SDS, 10 mM Tris, pH 7.5) containing protease and phosphatase inhibitors (Roche, cat. 04693124001 and 04906837001, Mannheim, Germany). Protein concentration was determined using a BCA protein assay (Pierce, cat. 23221). Equal amounts (40 µg/lane) of proteins were separated by SDS/PAGE (4–20% Mini-PROTEAN TGXTM Precast Gels, Bio-Rad Laboratories Inc., Hercules, CA, USA) and transferred to a nitrocellulose membrane (GE Healthcare, Piscataway, NJ, USA). Membranes were blocked with 1× Tris-buffered saline (TBS; Bio-Rad Laboratories Inc., Hercules, CA, USA) supplemented with 0.1% Tween-20 (Sigma-Aldrich, St. Louis, MO, USA) and containing 5% non-fat milk (Bio-Rad Laboratories Inc., Hercules, CA, USA) or 10% bovine serum albumin (Euroclone, Milan, Italy) for 1 h at room temperature (RT). Precision Plus Protein All Blue Standards (Bio-Rad Laboratories, Hercules, CA, USA) were used as molecular weight markers. The primary antibodies used in this work were: anti-AKT (all isoforms) antibody (Cell Signaling, cat. 9272, Danvers, MA, USA, 1:1000 dilution) or anti-p-AKT (Ser473) antibody (Cell Signaling cat. 4060, 1:1000 dilution). Anti-β-actin (mouse monoclonal antibody; Sigma-Aldrich, St. Louis, MO, USA) was used as a loading control. Blots were then incubated with horseradish peroxidase-conjugated secondary antibody (1:10,000, Vector Laboratories, Burlingame, CA, USA) for 1 h at RT. Signals were captured on a ChemiDoc™ Imaging System (Bio-Rad Laboratories, Hercules, CA, USA) using an enhanced chemiluminescence system (Super Signal Chemiluminescent Substrate, Thermo Fisher Scientific Inc. Waltham, MA, USA) and densitometric analyses were performed with Image Lab™ Touch Software (Bio-Rad Laboratories, Hercules, CA, USA). Total lysates were normalized using either stain-free technology (Bio-Rad Laboratories Inc., Hercules, CA, USA) or actin content. Phospho-AKT densitometric profiles were normalized versus total AKT. All experiments were carried out in triplicate and representative results are shown.

### 4.10. Scanning Electron Microscopy

NT2D1 were cultured as described above for 24 h. Samples then were fixed in 2.5% glutaraldehyde in cacodylate buffer (0.1 M pH 7.3) overnight, and post-fixed with 1% osmium tetroxide in cacodylate buffer (1 M). Then, samples were dehydrated with increasing ethanol percentage (30–90% in water for 5 min, twice at 100% for 15 min), dried in a critical point dryer (EMITECH K850), sputter coated with platinum–palladium (Denton Vacuum DESKV), and observed with a Supra 40 FE SEM (Zeiss).

### 4.11. Statistical Analyses

Statistical analyses have been carried out using Sigma Plot 11 Data Analyzer Software. Student’s t-test and ANOVA test (for multi-group comparison) were carried out. All quantitative data are presented as the mean ± standard error of the mean (SEM).

## Supplementary Materials

The following are available online at https://www.mdpi.com/1422-0067/21/22/8669/s1, Figure S1: Representative confocal images of actin organization (green) in NT2D1 cells during wound healing experiment. Lamellipodia and ruffles (dashed line) are more evident in HGF-treated cells (dotted line) respect to control. It is evident, especially in HGF-treated cells, that during movement, groups of cells remain connected via cell–cell junctions, a characteristic of the collective migration. Figure S2: Western blot analyses of phospho- and total AKT in NT2D1 cell line cultured in basal condition and after 30 min 5 µM LY294002, 40 ng/mL HGF and HGF + LY294002.

## Abbreviations

AEC	3-amino-9-ethylcarbazole
AKT	Protein kinase B
BCA	Bicinchoninic acid
BSA	Bovine serum albumin
c-MET	Mesenchymal–epithelial transition (HGF receptor)
DMEM	Dulbecco’s modified Eagle’s medium
EDTA	Ethylenediaminetetraacetic acid
FAs	Focal adhesions
FITC	Fluorescein Isothiocyanate
FBS	Fetal bovine serum
GCNIS	Germ cell neoplasia in situ
GFR	Growth factor reduced
HGF	Hepatocyte growth factor
HRP	Horseradish peroxidase
IGFR	Insulin-like growth factor receptor
PI3K	Phosphatidylinositol-3-kinase
PBS	Phosphate buffer saline
PDGFR	Platelet-derived growth factor receptors
PTEN	Phosphatase and tensin homolog
SDS	Sodium dodecyl sulfate
SE	Seminoma
S.E.M	Standard error of the mean
SEM	Scanning electron microscopy
SUM(I)	Sum of intensity
TGCTs	Testicular germ cell tumors
TRITC	Tetramethyl rhodamine iso-thiocyanate
VEGFR	Vascular endothelial growth factor receptor
