# Supplementary material for: The PI3K/AKT Pathway Is Activated by HGF in NT2D1 Non-Seminoma Cells and Has a Role in the Modulation of Their Malignant Behavior
Source: Int J Mol Sci. 2020 Nov 17;21(22):8669. doi: 10.3390/ijms21228669 (PMC7698414; doi:10.3390/ijms21228669)
Supplement: Supplementary file 1 [file ijms-21-08669-s001.zip › ijms-964578-supplementary.docx]

**Appendix A**


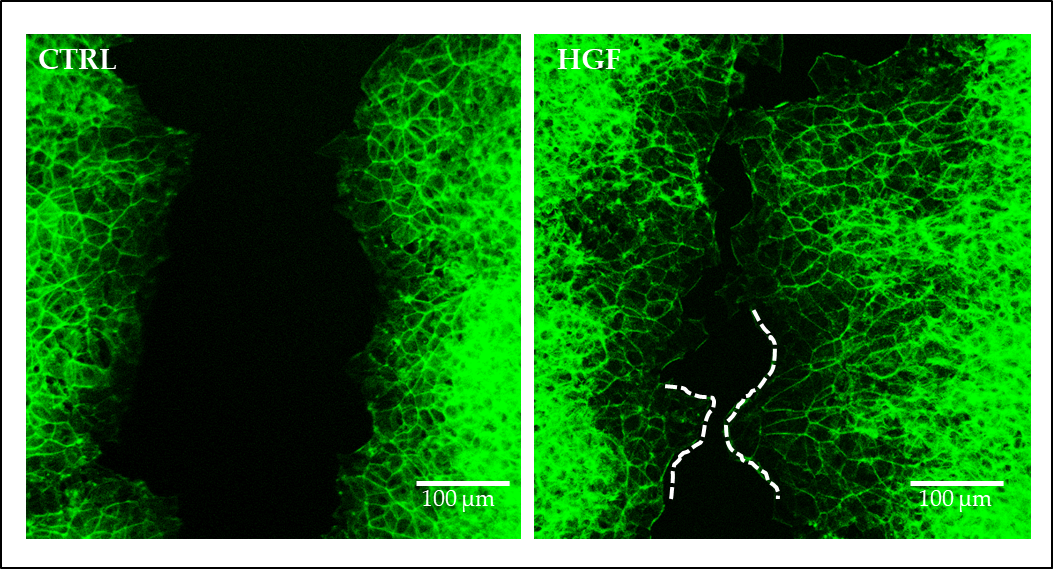


Figure S1: Representative confocal images of actin organization (green) in NT2D1 cells during wound healing experiment. Lamellipodia and ruffles (dashed line) are more evident in HGF-treated cells (dotted line) respect to control. It is evident, especially in HGF-treated cells, that during movement, groups of cells remain connected via cell–cell junctions, a characteristic of the collective migration.


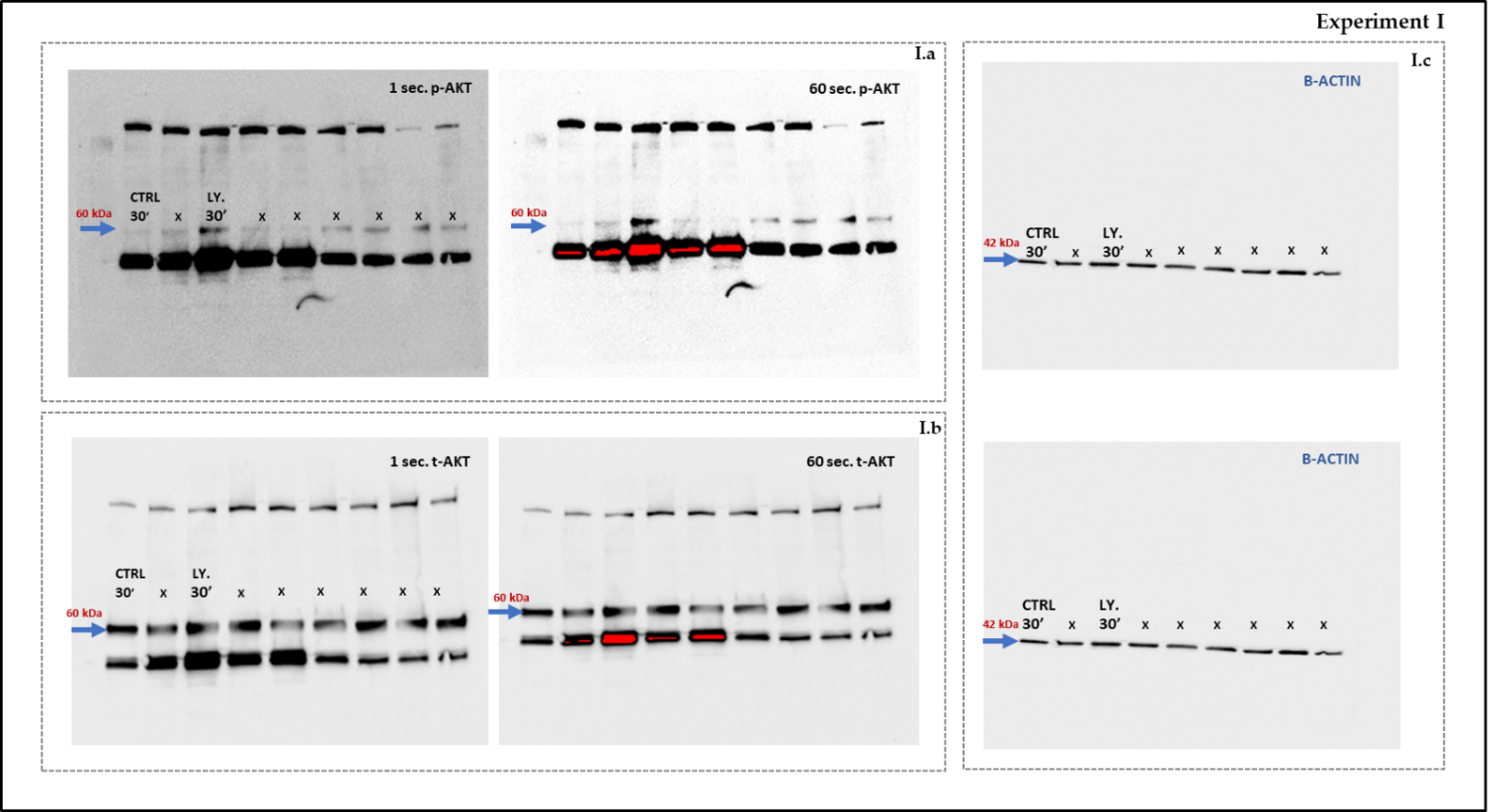


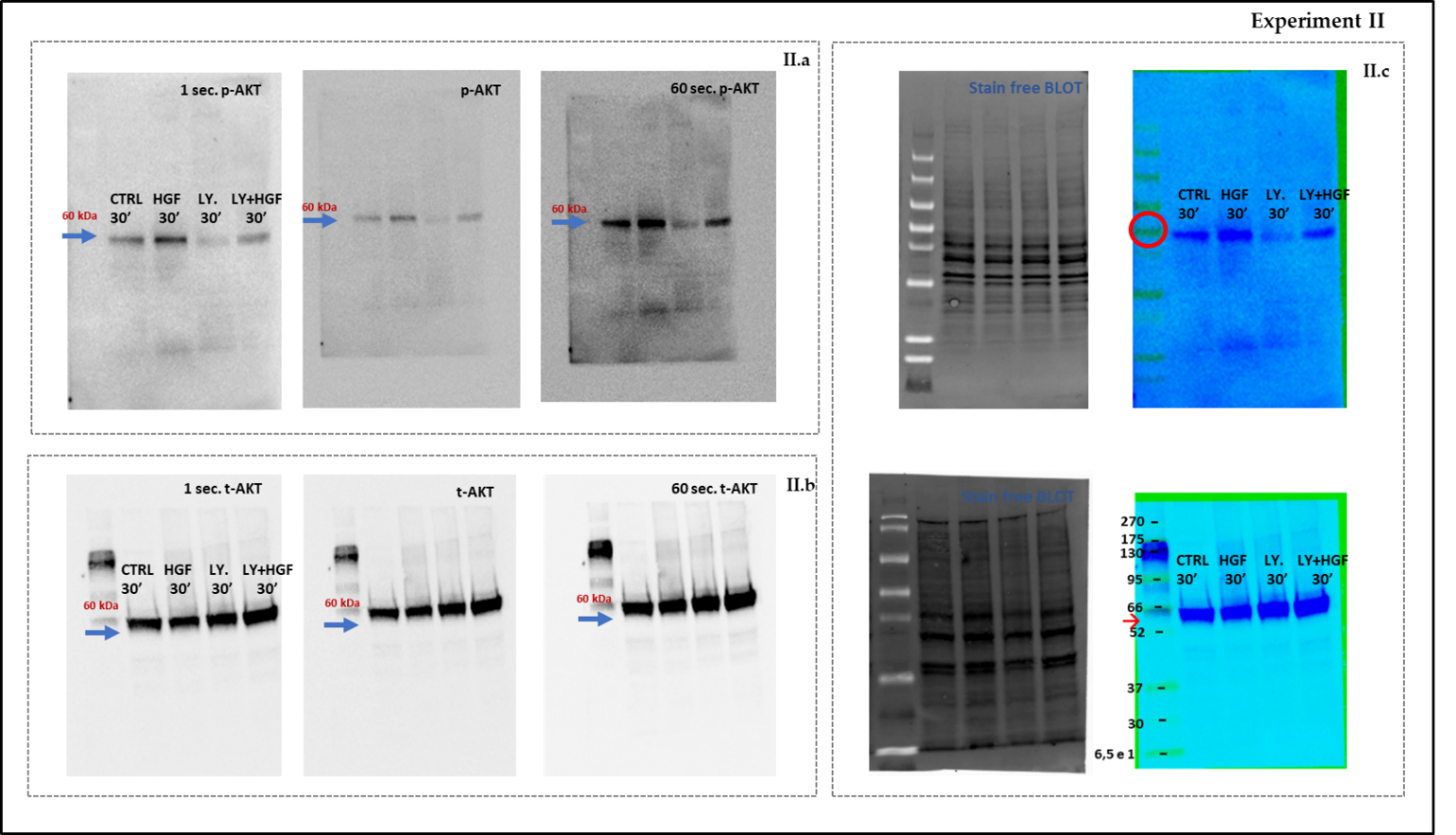


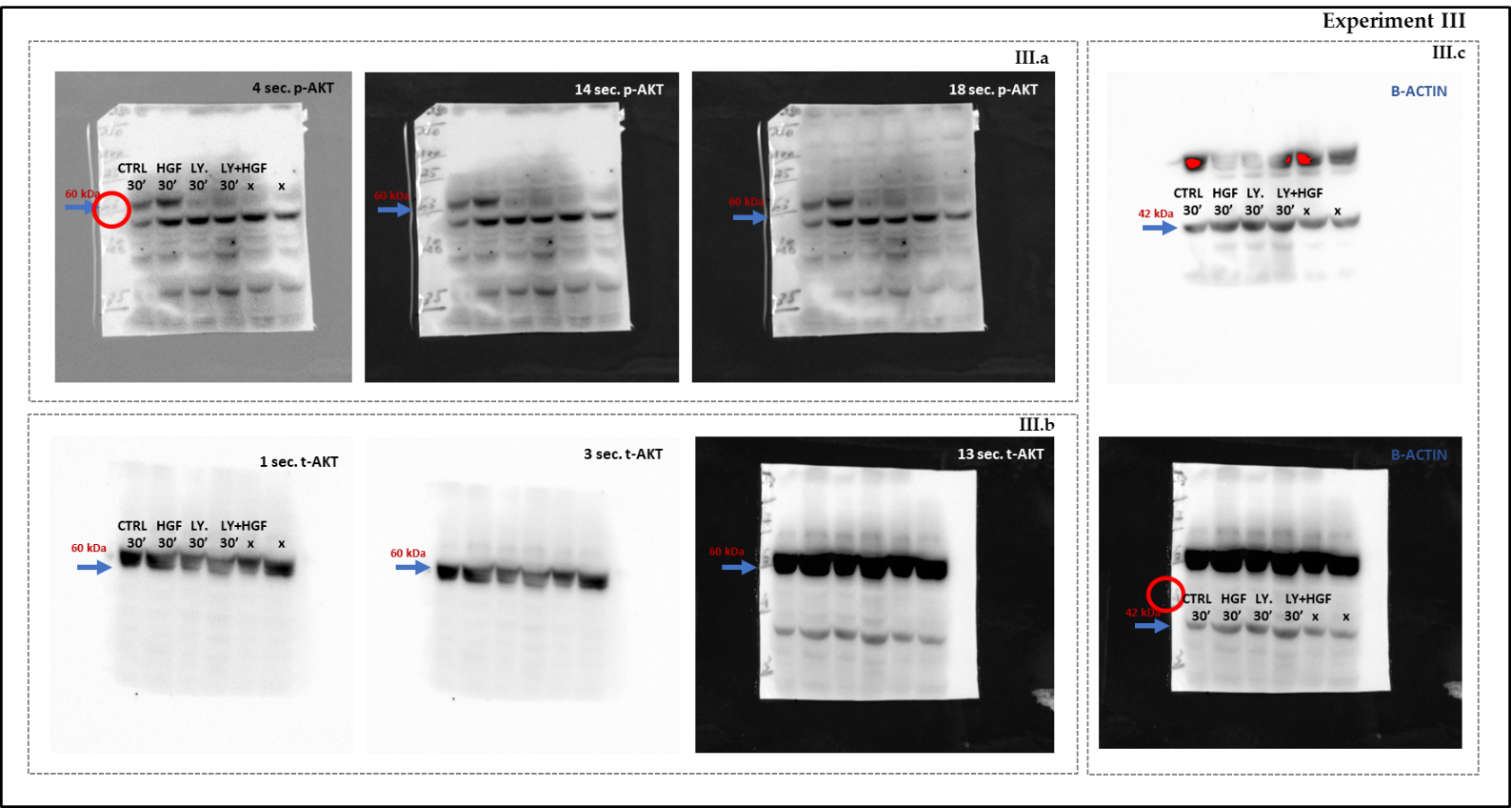


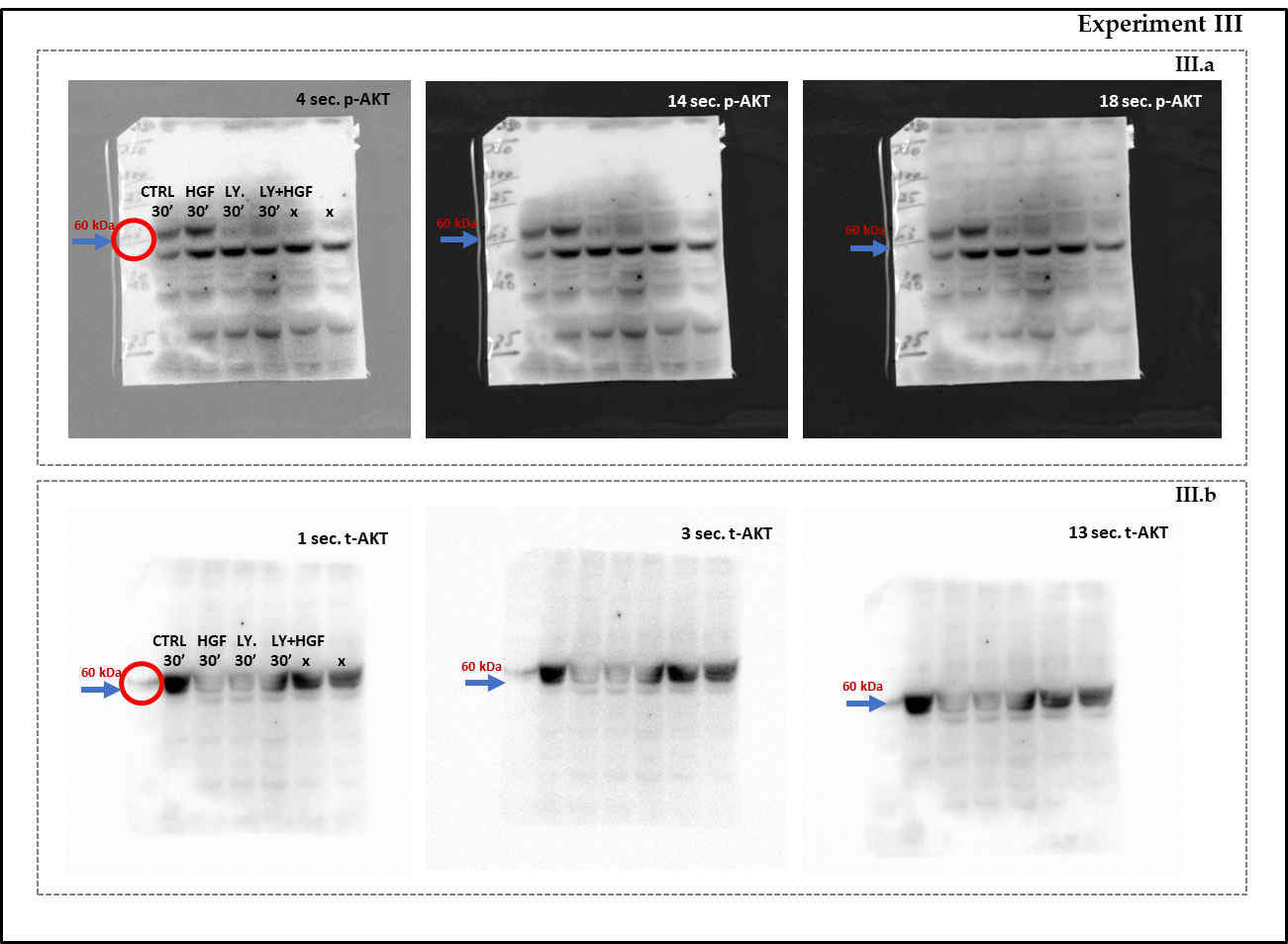


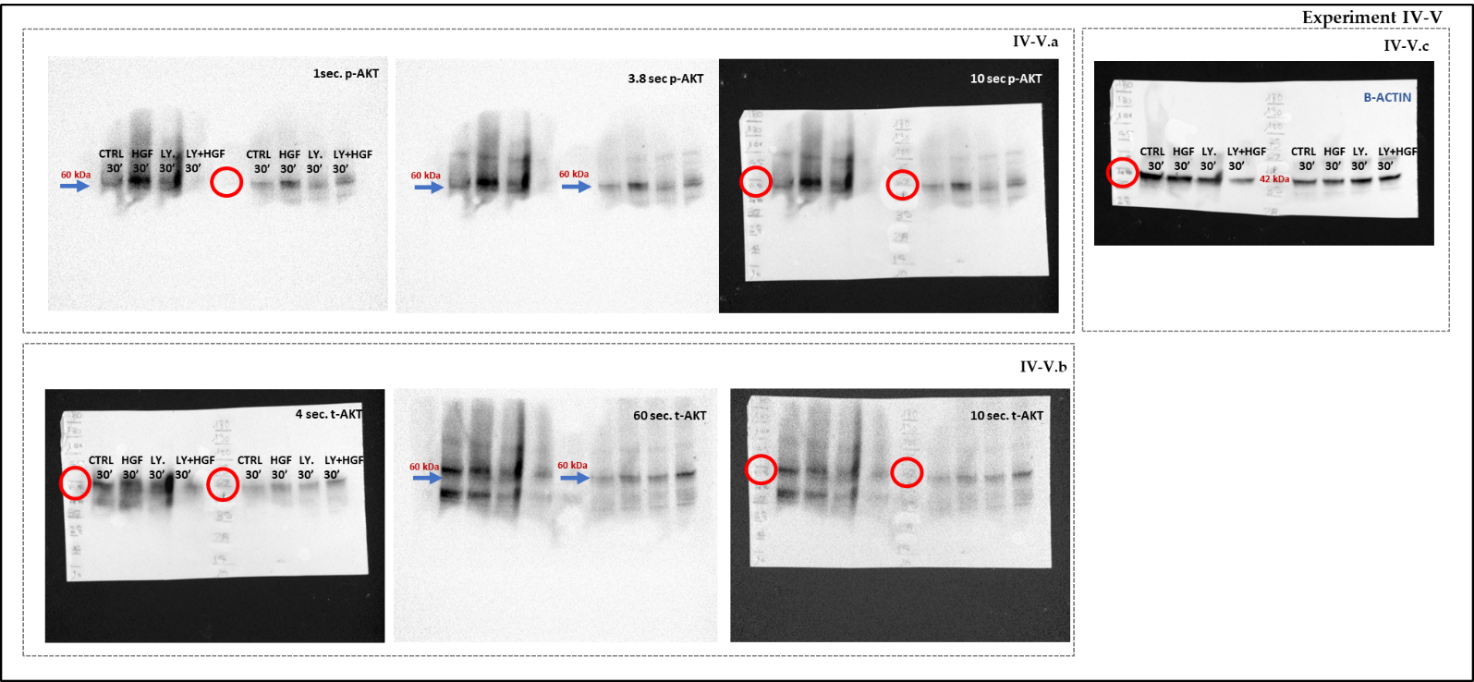


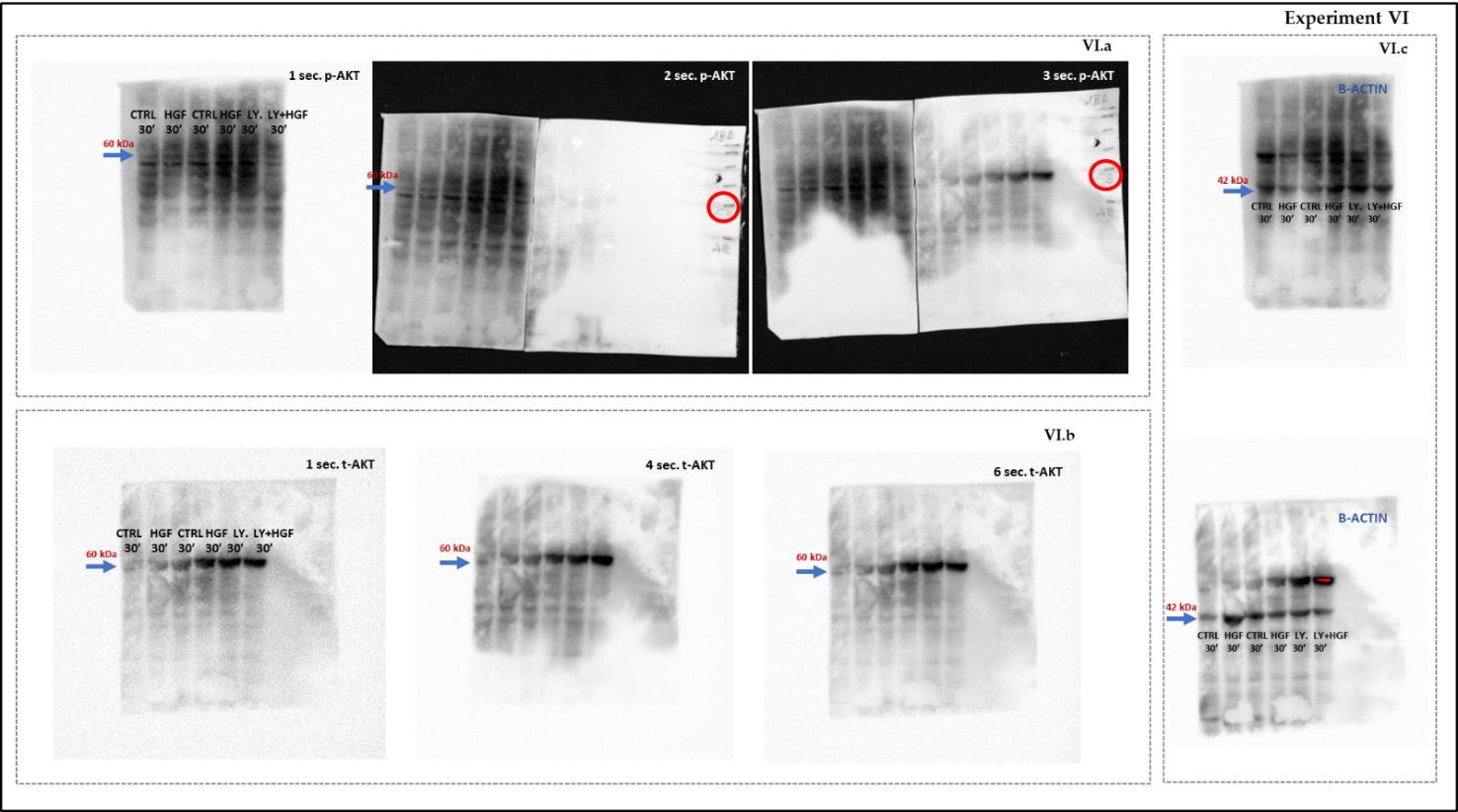


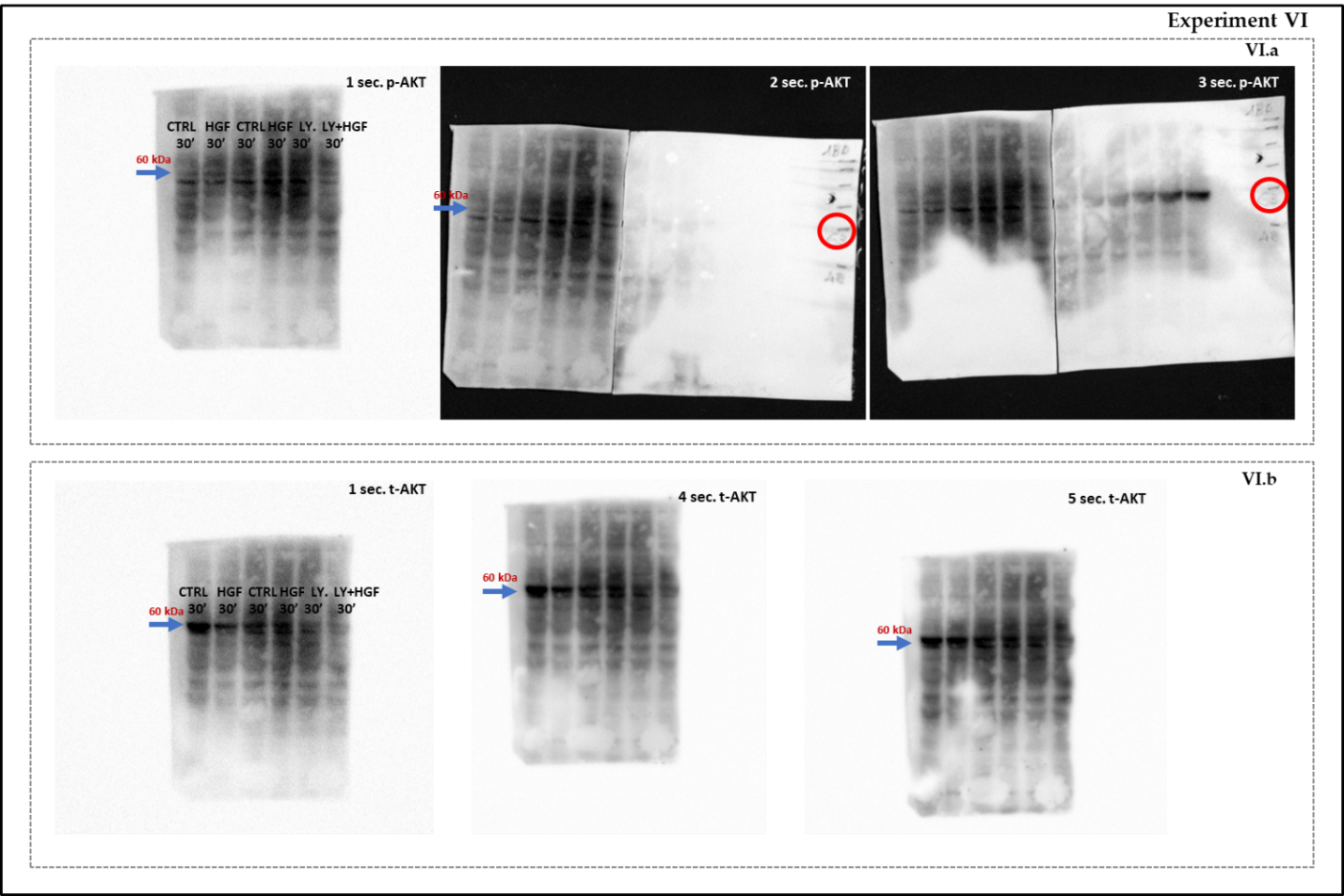


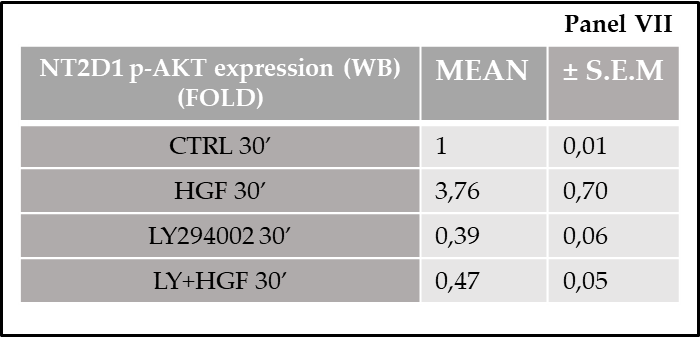


Figure S2. Western blot analyses of phospho- and total AKT in NT2D1 cell line cultured in basal condition and after 30 minutes 5uM LY294002, 40 ng/mL HGF and HGF + LY294002.

Representative images (Experiments I-VI) of phospho-AKT (I.a; II.a; III.a; IV.a; V.a; VI.a), total-AKT (I.b; II.b; III.b; IV.b; V.b; VI.b), and β-Actin (I.c; III.c; IV.c; V.c; VI.c) or stain free blot (II.c). All signals were recovered at different exposure times using ChemiDoc. Blu arrows indicate bands to the molecular weight of the protein of interest that were used for densiometric analyses. Bands at different molecular weight depend on the inbridization of the same filters with other antobodies not presented in the present manuscript. Panel VII: table illustrating the mean values +/- S.E.M. presented in the graph of Fig.2,IIb.
